# Effect of different liposomal bupivacaine concentrations in ultrasound-guided superior trunk block on postoperative analgesia and mobility: a randomized double-blind controlled trial protocol for shoulder arthroscopy

**DOI:** 10.1080/07853890.2026.2685345

**Published:** 2026-06-23

**Authors:** Yu Zhou, Hong-Zhang Ding, Xiao-Qian Li, Teng Zheng, Mao-Lin Ran, Ai-Ling Song, Yi-Min Qiu, Jin-Bao Li, Feng Chen

**Affiliations:** a Department of Anesthesiology, Shanghai General Hospital, Shanghai Jiao Tong University School of Medicine, Shanghai, China; b Department of Anesthesiology, Ruijin Hospital, Shanghai Jiao Tong University School of Medicine, Shanghai, China

**Keywords:** Liposomal bupivacaine, superior trunk block, postoperative pain, shoulder arthroscopy, protocol

## Abstract

**Background:**

Shoulder arthroscopy frequently causes severe postoperative pain that may impede recovery. Liposomal bupivacaine provides prolonged analgesia, and ultrasound-guided superior trunk block (STB) offers comparable analgesia to interscalene block with a lower risk of hemidiaphragmatic paralysis. However, the optimal concentration of liposomal bupivacaine for STB remains unknown.

**Methods:**

This randomized, double-blind, controlled trial will enrol 282 adult patients scheduled for elective arthroscopic rotator cuff repair. Patients will be randomly allocated (1:1:1) to receive ultrasound-guided STB with liposomal bupivacaine 66 mg (Group A), 44 mg (Group B) or 33 mg (Group C), each diluted to 10 mL. The co-primary outcomes are (1) rest pain Numeric Rating Scale (NRS) score at 48 h post-surgery and (2) cumulative oral morphine milligram equivalents (MME) consumption within 0–48 h after surgery. Secondary outcomes include rest pain NRS scores at 6, 24 and 72 h; motor function assessed by Muscle Balance Scale, Bromage score and American Shoulder and Elbow Surgeons (ASES) score at 6, 24, 48 and 72 h; and Quality of Recovery-15 (QoR-15) score at 24 and 48 h.

**Discussion:**

This study will provide evidence on the optimal concentration of liposomal bupivacaine for STB in arthroscopic shoulder surgery, aiming to achieve effective and prolonged analgesia without compromising shoulder mobility.

## Introduction

The shoulder’s wide range of motion makes it vulnerable to injuries, often causing pain, stiffness and functional loss that can significantly impact daily life. These injuries are common across all ages, especially in middle-aged, elderly and physically active individuals. Standard treatments include open repair, minimally invasive surgery and shoulder arthroscopy. Guided by Enhanced Recovery After Surgery (ERAS) principles, clinicians prioritize early rehabilitation. Shoulder arthroscopy, being less invasive than open surgery, minimizes trauma and enables faster postoperative recovery, making shortened recovery time a key focus in managing shoulder injuries [1].

However, shoulder arthroscopy utilizes pressurized joint irrigation and controlled hypotension to optimize visualization and haemostasis, yet these measures contribute to tissue swelling and notable postoperative pain [2]. Prolonged pain compromises patient satisfaction and recovery, with the risk of acute pain transitioning to chronic states. Effective analgesia is therefore vital to facilitate rehabilitation, enhance quality of life and fulfil the objectives of ERAS protocols.

Ultrasound-guided interscalene brachial plexus block (ISB) enables precise nerve targeting, minimizes injury to surrounding structures and provides effective intraoperative analgesia, underscoring its value within ERAS protocols for shoulder surgery. Studies demonstrate that combining ISB with general anaesthesia improves perioperative outcomes compared to general anaesthesia alone [3–7]. However, a single ISB provides only transient analgesia [8], and while continuous nerve blocks can improve pain control [9], they are prone to failure and complications [10].

Liposomal bupivacaine, a long-acting local anaesthetic providing pain relief for up to 72 h [11], was recently FDA-approved for ISB [12,13] to offer prolonged analgesia. Evidence suggests it can extend analgesia duration and reduce opioid consumption after brachial plexus blocks [14–16]. Nonetheless, ISB carries a significant risk of hemidiaphragmatic paralysis (HDP). The superior trunk block (STB) has been proposed as an alternative, providing comparable analgesia while markedly lowering HDP incidence [17–20].

In practice, high-concentration liposomal bupivacaine for brachial plexus blocks may cause prolonged motor blockade and potential phrenic nerve involvement, which could hinder early rehabilitation [21]. Although STB has been shown to preserve phrenic nerve function while providing comparable analgesia to interscalene block [20], no study to date has systematically evaluated different concentrations of liposomal bupivacaine specifically for STB. The optimal concentration that balances effective, prolonged analgesia with preserved motor function remains unknown. To address this research gap, this study aims to identify the optimal concentration of liposomal bupivacaine for STB in arthroscopic shoulder surgery, seeking to achieve effective and long-lasting analgesia while preserving shoulder joint mobility.

## Patients and methods

**Figure 1.**
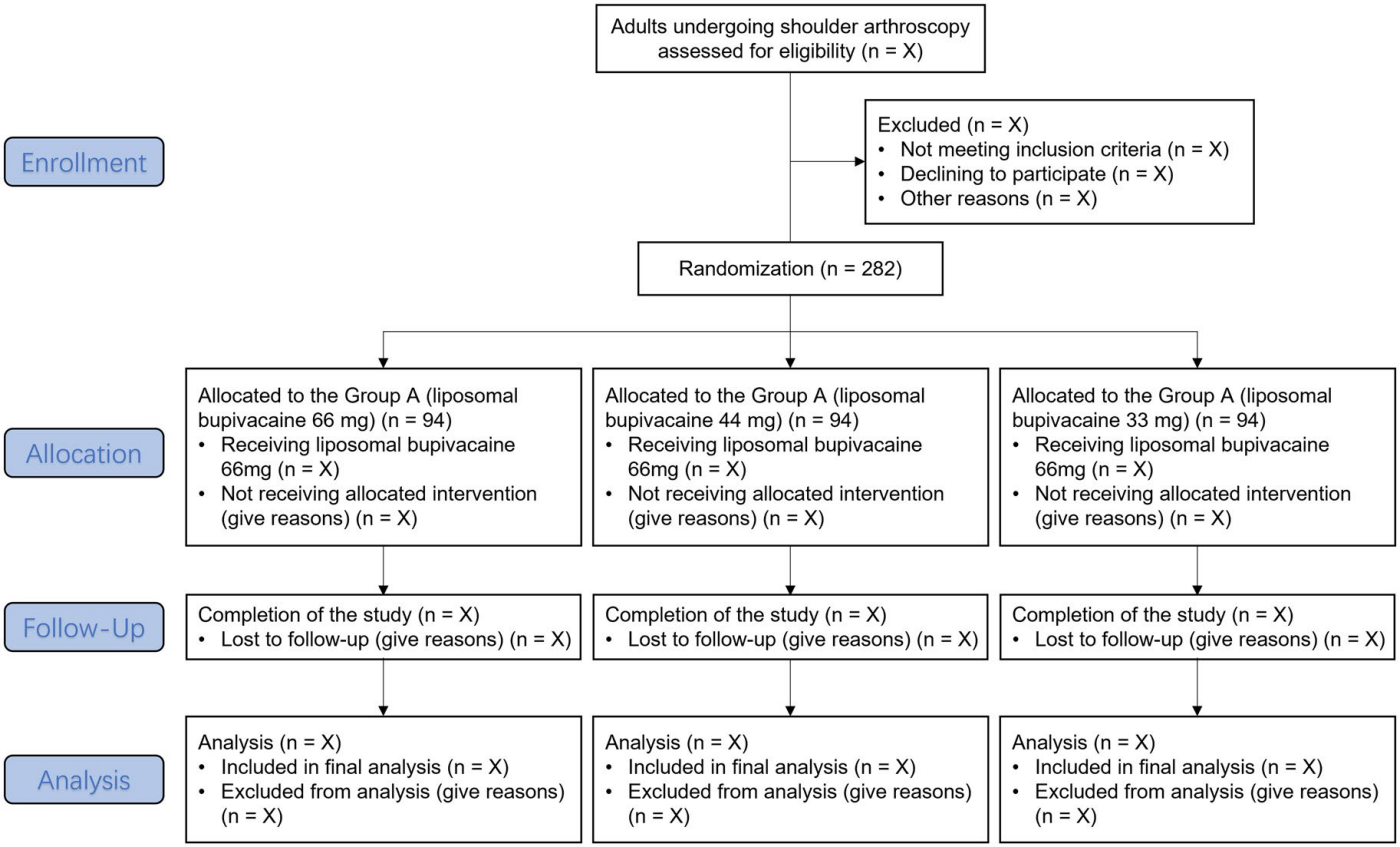
Study flow diagram.

This study will be conducted as a randomized, double-blind, controlled clinical trial. It was approved by the Ethics Committee of Shanghai General Hospital (Approval ID: 2024-209) on 27 November 2024 and the approved protocol was subsequently amended on 21 April 2026 (Approval ID: 2026-254). The protocol was registered with the Chinese Clinical Trial Registry (ChiCTR2400094688) on 26 December 2024 and the registry record was updated following the protocol amendment. We plan to recruit a total of 282 eligible patients from June 2026 to June 2027 (*n* = 94 per group) (Figure 1), all of whom will provide informed consent. The study will be conducted in compliance with the Declaration of Helsinki, and the protocol is reported per the Standard Protocol Items: Recommendations for Interventional Trials (SPIRIT) 2025 Statement: Updated Guideline for Protocols of Randomized Trials (Supplement 1)[22].

### Patients

The Inclusion criteria will be as follows: (1) adult patients aged ≥ 18 years old; (2) ASA classifications I-II; (3) scheduled to undergo elective arthroscopic rotator cuff repair; (4) demonstrate the ability to adhere to the study protocol and provide informed consent for participation in the clinical trial. Patients will be excluded if they meet any of these criteria: (1) history of upper limb neuropathy and chronic pain syndrome; (2) allergy to liposomal bupivacaine or other local anaesthetics (such as bupivacaine, ropivacaine or lidocaine); (3) severe respiratory disease; mental illness; cognitive impairment; drug or alcohol abuse; (4) long-term use of opioid (>3 months or Daily morphine intake >5 mg for 1 month); (5) contraindications to general anaesthesia or interscalene brachial plexus block; (6) pregnancy status.

### Randomization and blinding

An independent research coordinator, who will not be involved in the subsequent study, conducted the online randomization using a 1:1:1 allocation ratio and a permuted block size of 3. Subjects will be allocated to either Group A (liposomal bupivacaine 66 mg), Group B (liposomal bupivacaine 44 mg) or Group C (liposomal bupivacaine 33 mg) based on a random sequence (Figure 1). The doses were selected based on the following rationale. Notably, these dose selections remain partly exploratory, as robust pharmacodynamic and dose-response data for liposomal bupivacaine specifically in the superior trunk block (STB) are currently lacking. Nonetheless, the FDA-approved dose for interscalene block (ISB) is 133 mg. ISB targets the C5-C7 nerve roots at the interscalene groove. In contrast, STB is performed more caudally and laterally at the convergence of the C5 and C6 nerve roots, where they form the superior trunk of the brachial plexus. At this location, STB maintains comparable analgesia while allowing for lower local anaesthetic doses. Accordingly, we selected 66 mg (50% of the ISB dose) as the high-concentration group for STB. Second, 33 mg (25% of the ISB dose) was chosen to explore the minimal effective concentration. Third, 44 mg, the midpoint between 33 mg and 66 mg, was selected to identify the optimal balance between analgesia and motor function preservation. This trial is therefore designed to define the optimal concentration for STB. Allocation concealment will be ensured through the use of identical opaque sealed envelopes. An independent anaesthesia nurse at each study centre will be responsible for preparing the study medications. Given that varying concentrations of liposomal bupivacaine exhibit distinct appearances, we will maintain blinding for the surgeons and operating room staff by transferring the medication into identical opaque syringes, labelled solely with the patient’s identification number. The STB procedures will be conducted by a qualified anaesthesiologist, who will not participate in anaesthesia management or the supervision of postoperative care. All additional perioperative clinical care will adhere to the standard practices of the institution. Pertinent data will be documented in the trial case report form (CRF).

### Anaesthesia management

Prior to the induction of general anaesthesia, all participants will receive an ultrasound-guided STB. General anaesthesia will be induced using i.v. sufentanil 0.25 μg/kg, propofol 2 mg/kg and rocuronium 0.6 mg/kg. Anaesthesia will be maintained with desflurane at a concentration of 0.8–1.2 MAC, in conjunction with remifentanil administered at a rate of 0.2–0.3 µg/kg/min. Hemodynamic events, including hypotension (>30% decrease in mean blood pressure from baseline), hypertension (>30% increase in mean blood pressure from baseline), bradycardia (heart rate <50 beats/min) and tachycardia (heart rate >50 beats/min), fluid administration and other anaesthetic interventions will be managed by attending anaesthesiologists. A standardized Enhanced Recovery After Surgery (ERAS) protocol will be applied.

Rescue analgesia with intravenous flurbiprofen axetil (50 mg) will be administered as needed for pain with an Numeric Rating Scale (NRS) score ≥4. All analgesic management will be tailored to the patient’s perioperative condition and documented by the clinician.

### Study interventions

A single ultrasound-guided STB will be administered in the pre-anaesthesia room 1 hour preoperatively. The STB procedure will be performed by a qualified anaesthesiologist under sterile conditions. The patient will be placed in the supine position with the head turned slightly to the contralateral side. A high-frequency linear ultrasound transducer (6–13 MHz) will be positioned in the supraclavicular fossa with a transverse oblique orientation. The brachial plexus will be identified, and the superior trunk (formed by the C5 and C6 nerve roots) will be visualized as a hypoechoic structure lateral to the subclavian artery. A 22-gauge, 50-mm echogenic needle will be inserted using an in-plane approach from lateral to medial, with the needle tip placed adjacent to the superior trunk. Following negative aspiration, the assigned dose of liposomal bupivacaine (66, 44 or 33 mg) will be diluted with normal saline to a total volume of 10 mL and injected in 2–3 mL aliquots under real-time ultrasound guidance to ensure proper spread. Block success will be confirmed 15 min after injection by assessing sensory loss (cold test) and motor weakness (reduced strength of deltoid and biceps muscles) compared with the contralateral side. Following the block procedure, patients will undergo continuous electrocardiogram (ECG) monitoring for 1 hour to enable immediate management of any serious adverse events. Known side effects of STB include nausea, vomiting, constipation, tachycardia, bradycardia, dizziness, headache, somnolence and hypoesthesia. The most serious risk is arrhythmia due to inadvertent intravascular injection, which ultrasound guidance helps to mitigate. Mild adverse events will not require treatment; moderate-to-severe events will receive appropriate pharmacologic intervention. If a severe event occurs after 50% of the dose has been administered, the injection will be discontinued immediately. Acute allergic reactions will be managed according to standard institutional protocols. All adverse events will be fully documented in the case report form.

### Primary and secondary outcomes

The co-primary outcomes will be (1) Rest pain NRS score at 48 h post‑surgery; (2) Cumulative opioid consumption within 0–48 h after surgery, converted to oral morphine milligram equivalents (MME). Secondary outcomes will be (1) Rest pain NRS scores at 6 h, 24 h and 72 h post‑surgery; (2) Muscle Balance Scale (MBS) ratings at 6 h, 24 h, 48 h and 72 h; (3) Motor nerve block assessed by the Bromage score at 6 h, 24 h, 48 h and 72 h [23]; (4) American Shoulder and Elbow Surgeons (ASES) functional score at the same intervals (Table 1); (5) Quality of Recovery-15 (QoR-15) score at 24 h and 48 h post‑surgery.

**Table 1.** The American shoulder and elbow surgeons (ASES) score.

Activity	Unable to do Score = 0	Very difficult to do Score = 1	Somewhat difficult Score = 2	Not difficult Score = 3
Do usual work				
Do usual sport				
Put on a coat				
Sleep on your painful or affected side				
Wash back/do up bra in back				
Manage toileting				
Comb hair				
Reach a high shelf				
Lift 4.5kg above shoulder				
Throw a ball overhand				
Total scores				

A patient-reported subjective rating system was employed to assess the score, comprising two components: pain (50%) and functional capacity (50%), with a total possible score of 100. Higher scores indicate superior shoulder function.

Specific scoring method: pain score = (10-NRS score) *5; Functional score = total score of 10 items *5/3.

ASES score = pain score + function score.

Although liposomal bupivacaine provides analgesia lasting up to 72 h, its most stable and clinically consistent effect occurs within the first 48 h after surgery. This period also represents the critical window for early postoperative rehabilitation, opioid consumption and acute pain control. Therefore, 48 h was selected as the primary time point to capture the most clinically meaningful analgesic difference among groups, while 72 h was retained as a secondary outcome to assess the extended duration of action.

NRS scores range from 0 (no pain) to 10 (worst imaginable pain). Mild pain (1–3) is non‑disruptive to sleep, moderate pain (4–6) mildly disrupts sleep and severe pain (7–9) prevents or awakens from sleep.

The MBS assesses elbow and wrist flexion strength using a 5‑point scale: 0 (no muscle activity), 1 (slight movement), 2 (movement against gravity only), 3 (movement against resistance with reduced strength) and 4 (strong movement against resistance).

The ASES scoring system is utilized to assess the functional performance of the shoulder joint on the affected side (Table 1).

The QoR-15 score assesses the quality of postoperative recovery. It consists of 15 items, each scored from 0 to 10 (0 = completely disagree/very poor condition; 10 = completely agree/excellent condition). The total score ranges from 0 to 150, with higher scores indicating better postoperative recovery quality (Table 2).

**Table 2.** The quality of recovery-15 (QoR-15) score.

Part	Item no.	Item description	Score (0–10)
**A. How have you felt in the past 24/48 hours?**			
A1	1	Breathing easily	
A2	2	Good appetite	
A3	3	Feeling energetic	
A4	4	Good sleep quality	
A5	5	Being able to perform personal hygiene and toileting independently	
A6	6	Being able to communicate with family or friends	
A7	7	Receiving support from doctors and nurses	
A8	8	Being able to return to work or perform routine household tasks	
A9	9	Feeling comfortable and in control of emotions	
A10	10	Feeling generally better	
**B. Have you experienced any of the following in the past 24/48 hours?**			
B1	11	Moderate pain	
B2	12	Severe pain	
B3	13	Nausea or vomiting	
B4	14	Feeling worried or anxious	
B5	15	Feeling sad or depressed	
Total		**Sum of all items (0–150)**	

### Data collection

Table 3 outlines the schedule of enrolment, interventions and data measurements in accordance with the SPIRIT guidelines. Demographic data, ASA status, comorbidities, preoperative medications and the age-adjusted Charlson Comorbidity Index (aCCI) are scheduled to be collected prior to anaesthesia. Surgical, anaesthesia and perioperative data, including hemodynamic events, will be documented *via* an electronic system.

**Table 3.** Schedule of patient enrolment, study interventions, and outcome assessment according to SPIRIT statement.

			Study period				
Timepoint	Enrollment Preoperative visit	Allocation Before surgery	Post-allocation				
			Pre-anesthesia room	6 h	24 h	48 h	72 h
Enrollment							
Inclusion criteria	×						
Exclusion criteria	×						
Written informed consent	×						
Baseline data	×						
Randomization		×					
Allocation		×					
Study interventions							
Group A			**×**				
Group B			**×**				
Group C			**×**				
Outcome assesment							
NRS score				**×**	**×**	**×**	**×**
MME						**×**	
MBS score				**×**	**×**	**×**	**×**
Bromage score				**×**	**×**	**×**	**×**
ASES score				**×**	**×**	**×**	**×**
QoR-15 score					**×**	**×**	

To minimize reporting errors in the study, only investigators who will be blinded to the study design and group assignment will conduct the postoperative follow-up, which will include assessments of postoperative pain and motor block. Outcome measures will be documented and evaluated in accordance with the procedures outlined in the blinded section. An independent Data Monitoring Committee (DMC), composed of an attending orthopaedic surgeon, an attending anaesthesiologist, a pharmacist and a statistician, will oversee the conduct of the study.

### Sample size estimation

Sample size calculation was performed using PASS 2021 software (NCSS, USA) based on a previous study [24,25] and our preliminary data, with the goal of ensuring adequate power for both co-primary endpoints.

For the 48-hour moderate-to-severe pain incidence (dichotomized NRS): Based on our preliminary dose-finding results, the incidence of moderate-to-severe pain at 48 h was assumed to be 15% for Group A (66 mg), 25% for Group B (44 mg) and 35% for Group C (33 mg). Without optimized long-acting regional analgesia, approximately 74.3% of patients experience moderate-to-severe pain following arthroscopic rotator cuff repair [24,25]. Assuming 1-β = 0.8, α = 0.05 and *R* = 1, 84 participants per group are required.

For the co-primary outcome of cumulative 0–48 h opioid consumption (MME, continuous): Based on our preliminary data and published literature, the mean cumulative MME within 0–48 h was assumed to be 20 mg (SD = 12) in Group A, 35 mg (SD = 12) in Group B and 50 mg (SD = 12) in Group C. Using one-way ANOVA for three-group comparison with α = 0.05 and power = 0.8, 72 participants per group are required.

The larger requirement (84 per group) was adopted to provide ≥80% power for each co-primary endpoint. To allow for a 10% dropout rate, we will enrol 94 patients per group, resulting in a total sample size of 282 participants.

### Statistical analysis

Normally distributed continuous variables will be presented as mean ± standard deviation (SD), non-normally distributed variables as median (interquartile range, IQR) and categorical variables as number (percentage).

For outcomes assessed at multiple time points, a linear mixed model for repeated measurements (MMRM) will be employed, with fixed effects including group, time point and the group × time interaction, patient as a random intercept, and pairwise comparisons between groups adjusted using Tukey’s correction. Missing data in repeated-measures analyses will be handled under the assumption of missing at random (MAR) using maximum likelihood estimation. The MMRM model naturally accommodates missing data without pre-imputation. A sensitivity analysis using multiple imputation will be conducted to test the robustness of results.

For single time point analyses, normally distributed continuous variables will be compared using one-way ANOVA, followed by post‑hoc pairwise comparisons with Tukey’s correction; non-normally distributed variables using the Kruskal‑Wallis test; and categorical variables using the chi‑square test or Fisher’s exact test as appropriate. Multivariable linear regression will be used to adjust for potential confounding factors, including age, sex, body mass index (BMI), baseline NRS score and surgical duration. A two-sided *P* value < 0.05 will be considered statistically significant.

For the trial to be declared positive regarding the primary hypothesis, both co-primary endpoints must reach statistical significance. Specifically, for at least one pairwise comparison, a statistically significant difference must be observed in both the 48-hour resting pain NRS score and the cumulative 0–48 h MME consumption, after applying Tukey’s correction for multiple comparisons within each endpoint. No additional alpha adjustment (e.g. Bonferroni) will be applied across the two co-primary endpoints. According to ICH E9 guidelines for co-primary endpoints, when a trial requires statistical significance on all co-primary endpoints to declare a positive result, the family-wise error rate is inherently controlled without further adjustment. This principle applies here, as both endpoints must demonstrate improvement to define a clinically successful analgesic regimen.

The primary analysis will follow the intention-to-treat (ITT) principle. The ITT population includes all randomized patients who received the study intervention and had at least one post-baseline outcome assessment. As a supportive analysis, a per-protocol (PP) sensitivity analysis will be performed to assess the robustness of the findings. The PP population includes patients without major protocol deviations.

Statistical analyses will be conducted using the SPSS 20.0 software (IBM SPSS, Chicago, IL, USA).

## Discussion

This study investigates the optimization of regional analgesia within ERAS protocols for shoulder arthroscopy, focusing on the balance between prolonged pain control and functional recovery. The shift from the traditional ISB to the STB is primarily motivated by the imperative to reduce iatrogenic morbidity, specifically HDP. By selectively targeting the superior trunk, STB significantly mitigates the risk of phrenic nerve palsy [17–20], enhancing patient safety-a cornerstone of the ERAS philosophy. This safety profile makes STB particularly advantageous for patients with pre-existing pulmonary compromise.

The incorporation of liposomal bupivacaine addresses the well-documented limitation of short-lived analgesia from single-injection nerve blocks. Its sustained-release formulation has demonstrated superiority over conventional local anaesthetics in providing pain relief during the critical 24 to 48-hour postoperative window and in reducing opioid requirements [14–16]. However, its extended duration of action presents a unique challenge in the context of peripheral nerve blocks: the potential for disproportionately long-lasting motor blockade. In shoulder surgery, where early passive and active motion is crucial for recovery, prolonged motor weakness could paradoxically impede rehabilitation progress and patient satisfaction, counteracting ERAS goals.

Existing literature offers limited guidance on employing liposomal bupivacaine in the STB approach. While its efficacy in ISB is established [14–16], the pharmacokinetics and optimal dosing for the more focused STB technique require dedicated evaluation. The central question is not merely whether it works, but at what concentration it provides an optimal sensory-motor dissociation – sufficient analgesia without undue motor impairment. This study directly addresses this gap by systematically evaluating different concentrations to define this therapeutic window.

The clinical implications of defining an optimal concentration are substantial. A successful regimen would yield a multimodal analgesic strategy that synergizes safety (low HDP risk of STB), efficacy (prolonged analgesia from liposomal bupivacaine) and functionality (preserved motor function for early mobilization). This aligns perfectly with the holistic aims of ERAS, potentially improving recovery quality, reducing opioid-related side effects and increasing patient satisfaction after shoulder arthroscopy.

This study has limitations. It primarily assesses pharmacodynamic outcomes (analgesia and motor function); larger-scale studies are needed to confirm the safety profile and precisely quantify the incidence of rare complications with this specific combination. Furthermore, the ideal concentration may vary based on surgical complexity (e.g. subacromial decompression vs. rotator cuff repair), suggesting a need for procedure-specific refinements in future research. Long-term follow-up would also be valuable to assess the impact of this analgesic approach on the development of chronic postoperative pain and final functional outcomes.

## Supplementary Material

Supplement 1 SPIRIT 2025.docx

## Data Availability

The full protocol, participant-level dataset, statistical plan and informed consent materials can be available *via* contacting the corresponding author after the formal publication of this trial.
